# A272 EFFECTS OF EMPAGLIFLOZIN ON CHEMICALLY INDUCED COLITIS IN A MOUSE MODEL ARE MODULATED BY SEX AND DIETARY INTERACTIONS

**DOI:** 10.1093/jcag/gwad061.272

**Published:** 2024-02-14

**Authors:** K Madsen, B Villaflor, N Hotte, A Thiesen, C Cheng, T Omeltchenko

**Affiliations:** University of Alberta, Edmonton, AB, Canada; University of Alberta, Edmonton, AB, Canada; University of Alberta, Edmonton, AB, Canada; University of Alberta, Edmonton, AB, Canada; University of Alberta, Edmonton, AB, Canada; University of Alberta, Edmonton, AB, Canada

## Abstract

**Background:**

High sugar diets have been shown to dramatically increase disease severity in mouse models of colitis. Empagliflozin (EMPA) is a highly selective sodium glucose cotransporter-2 (SGLT2) inhibitor that is used therapeutically as an antihyperglycemic agent in the management of type 2 diabetes. In human trials EMPA treatment exerts potent anti-inflammatory effects independently of glycemic control. Further, we have previously demonstrated in a genetic mouse model of colitis that EMPA treatment was highly effective in improving colonic inflammation. Based on these findings, we hypothesized that EMPA treatment may also be effective in mitigating disease severity in chemically induced colitis in mice fed a high sugar diet.

**Aims:**

The aim of this study was to examine the effects of treatment with EMPA on chemically induced colitis in mice fed a high sugar diet

**Methods:**

At 6-8 weeks of age, wild-type 129/SvEv mice were placed on chow (CH) or high sugar diet (HS) (50% sucrose: AIN76A) ± EMPA (10mg/kg). After two days on the diet, mice were administered dextran sodium sulfate (DSS) for 5 days in drinking water followed by water alone for 2 days (n=4-6 mice for all groups). Disease activity index (DAI) was calculated daily from animal weight change, stool consistency, and stool hemoccult. As a measurement of degree of healing, colonic tissues were evaluated at day 9 for histological changes, weight-to-length ratio, and cytokine levels by ELISA. As a measure of EMPA functionality, urinary and blood glucose levels were measured.

**Results:**

Mice on the HS diet demonstrated increased susceptibility to colitis compared with chow fed mice with increased colonic levels of IL-1β, weight loss, and DAI. Significant interactions between sex and diet were seen in responses to EMPA treatment. EMPA treatment did not alter severity of colitis but did significantly delay healing in male mice on both the HS and chow diets as evidenced by increased DAI and increased enterocyte injury with increased levels of lamina propria neutrophils and lymphocytes. EMPA treatment did not increase disease severity in females on either diet. No differences were seen in colonic TNFα, IL-10, IL-12p70, or IL-22 between any of the groups. Urinary glucose levels were elevated in mice receiving EMPA and significantly higher in males compared with females (pampersand:003C0.05). EMPA treatment did not alter fasting blood glucose levels.

**Conclusions:**

A high sugar diet strongly exacerbates disease severity in a sex-dependent manner in a chemically induced model of colitis. Further, contrary to our hypothesis, EMPA treatment did not improve colitis in females and worsened disease in males suggesting that the documented anti-inflammatory effects of EMPA may be condition dependent.

**Funding Agencies:**

CCC

